# Strain‐Dependent Fermentation of Banana Pulp by Lactic Acid Bacteria Modulates Bioactive Metabolites and Supports Plant‐Based Ingredient Development

**DOI:** 10.1002/fsn3.72577

**Published:** 2026-11-11

**Authors:** Sara Morandi, Gabriele Rocchetti, Claudia Cortimiglia, Daniela Bassi

**Affiliations:** ^1^ Department for Sustainable Food Process Università Cattolica del Sacro Cuore Cremona Italy; ^2^ Department of Animal Science, Food and Nutrition Università Cattolica del Sacro Cuore Piacenza Italy; ^3^ Department for Sustainable Food Process Università Cattolica del Sacro Cuore Piacenza Italy

**Keywords:** freeze‐drying, *Lactiplantibacillus plantarum*, phenolic compounds, UHPLC‐HRMS, untargeted metabolomics, *Weissella cibaria*

## Abstract

Lactic fermentation of fruit matrices can yield functional ingredients; yet the potential of banana pulp as a substrate remains poorly explored. This study characterized the fermentation of banana pulp purée by three lactic acid bacteria (LAB) species, *Lactiplantibacillus plantarum* (P), *Levilactobacillus brevis* (B), and *Weissella cibaria* (W), applied as single and mixed starter cultures. At strain level, the three species were assessed for exopolysaccharide (EPS) production, antifungal activity, carbohydrate utilization and cell growth in the plant matrix. All the strains reached from 8 to 9 log_10_ CFU/mL within 24 h. *W. cibaria* was the only EPS producer but acidified slowly and remained closest to the control, whereas *L. plantarum* gave the strongest and fastest acidification (pH from ~4.8–5.0 to ~3.4–3.6), highest cell densities, and earliest reduction of *Enterobacteriaceae* (24 h). Freeze‐drying applied on the fermented banana pulp preserved 6.93–7.91 log_10_ CFU/mL across all formulations. Untargeted UHPLC‐HRMS annotated 433 metabolites and provided semi‐quantitative values showing that fermentation, particularly in mixed cultures, promoted the accumulation of flavonoids, ascorbic acid‐related metabolites, folic acid (up to 4.8 μg Eq./100 g) and 3‐indolelactic acid, and a reduction of flavanols and 5‐MTHF. PB combined rapid acidification with the highest cell density in 24 h and folate levels closest to the control (106.6 vs. 106.3 μg Eq./100 g), while PBW reached the highest flavanol content (105.2 vs. 65.3 μg Eq./100 g). Overall, LAB fermentation modified the metabolic profile of banana pulp, supporting its further evaluation as a potential functional ingredient.

## Graphical abstract

Banana pulp purée is fermented with single and mixed cultures of *Lactiplantibacillus plantarum*, *Levilactobacillus brevis* and *Weissella cibaria* . Fermentation modulates the metabolite profile in a strain‐dependent manner, increasing flavonoids, flavones, ascorbic acid‐related metabolites and folic acid, and decreasing flavanols and 5‐methyltetrahydrofolate. Freeze‐drying preserves viable lactic acid bacteria, supporting the fermented pulp as a potential plant‐based ingredient.

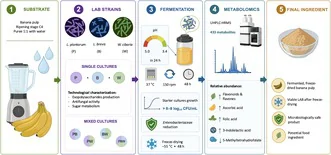

## 1 Introduction

In recent years, increasing consumer awareness of the relationship between diet and health has driven substantial interest in foods able to provide physiological benefits beyond basic nutritional requirements. These products, commonly referred to as functional foods (Fekete et al. 2025), are defined as foods that, when consumed as part of a normal diet, provide physiological benefits beyond their nutritional content (Aronson 2017).

Among the strategies available to obtain such products from plant raw materials, fermentation with lactic acid bacteria (LAB) is well established, since it can improve both nutritional value and microbial stability through natural acidification (Lahiri et al. 2022). LAB are crucial in the field of fermented foods derived from both animal and plant sources. They efficiently convert carbohydrates into lactic acid, producing minor amounts of other organic acids that lower pH, and enhance shelf life and stability. Their metabolic activity also improves the technological and sensory properties of fermented foods, such as texture, digestibility, and flavor (Sabater et al. 2020). Recent work stresses that these outcomes are substrate‐, strain‐ and process‐dependent rather than general, and that omics approaches are increasingly used to follow how starter cultures reshape plant matrices (Sam and Aryee 2026).

Among the plant matrices suitable for this purpose, bananas are of particular interest: they are among the most cultivated fruits worldwide, with an annual production exceeding 115 million tonnes (Kumari et al. 2022). From a compositional and technological standpoint, they represent an excellent substrate for fermentation, owing to their high content of readily fermentable carbohydrates, favorable natural acidity, and the relatively soft structure of the pulp, which facilitates microbial access to sugars and other nutrients. Experimental studies have indeed highlighted the potential of banana pulp to support microbial fermentative activity, with performance strongly influenced by the degree of ripeness, further highlighting the possible use of banana purée as a suitable substrate for lactic fermentation (Gallo et al. 2021). The pulp is an excellent source of carbohydrates (sucrose, glucose, fructose), vitamins, minerals, and several bioactive compounds, including essential phytonutrients whose concentration depends on the variety and degree of ripeness (Yap et al. 2017). During ripening, the starch naturally present in bananas is progressively degraded into simpler, soluble sugars, leading to a decrease in starch content and a concomitant increase in sweetness. Ripened bananas contain around 23 g of carbohydrates per 100 g, mostly in the form of sugars. The fruit also provides both soluble fibers (pectin, hemicellulose) and insoluble fibers (cellulose, lignin, resistant starch), as well as a natural acidity (pH ≈4.5) due to organic acids such as ascorbic, citric, malic, and oxalic acids, which contribute to both flavor and preservative properties (Aboul‐Enein et al. 2016).

The ripening stage is generally classified using the Von Loesecke scale, which defines seven color stages ranging from fully green (C1 = unripe) to yellow with brown spots (C7 = overripe) (Gomes et al. 2013). Usually, Stage 1–2 is rarely used for direct consumption or fermentation due to their limited flavor development and hard structure. Conversely, overripe fruits at Stage 7 tend to exhibit excessive softening, enzymatic browning, and higher susceptibility to microbial spoilage, making them less suitable for controlled fermentation processes. Beyond its compositional suitability, the lactic fermentation of fruit matrices is also of interest within the broader effort to extend the use of plant‐based raw materials along the food chain (Sabater et al. 2020).

The most common strains used for fermentation of vegetable substrates are *Streptococcus thermophilus* , *Lactococcus lactis* , *Leuconostoc* spp., *Lactobacillus* spp., but also strains belonging to *Pediococcus*, *Oenococcus*, and *Weissella* genus are gaining importance in plant based fermented products (Yang et al. 2024). When selecting a starter culture, it is essential to evaluate the metabolic traits of each strain in relation to the desired technological and functional outcomes. Among the various LAB species applied in plant‐based fermentations, *Lactiplantibacillus plantarum*, *Levilactobacillus brevis*, and *Weissella cibaria* are of particular interest due to their adaptability to plant‐origin substrates and their potential to enhance product quality. *L. plantarum* is a versatile and acid‐tolerant species widely applied in the fermentation of plant‐based matrices, thanks to its ability to efficiently metabolize a broad range of carbohydrates and adapt to complex vegetable substrates (Arena et al. 2016). *L. brevis* is a heterofermentative bacterium, frequently used as a probiotic culture (Ramos et al. 2013; Rönkä et al. 2003), that can utilize diverse oligosaccharides inhibiting undesirable microorganisms and supporting gut‐associated beneficial microbes (Bhola and Bhadekar 2024; Tang et al. 2022; Tebyanian et al. 2017). *W. cibaria* is known for its capacity to produce exopolysaccharides (EPS) in plant‐based fermentations (Nguyen et al. 2024; Zhu et al. 2018).

Among the technological traits relevant to plant‐based fermentation, the ability to synthesize EPS is of particular interest. These extracellular polymers increase viscosity and water‐holding capacity, and can improve texture and stability in fruit and vegetable matrices, where the loss of structure during processing is a common limitation (Guérin et al. 2020). EPS synthesis is markedly strain‐dependent and influenced by the carbon source available: in *Weissella* and related genera, glucansucrase and fructansucrase activities convert sucrose into glucans and fructans, so that production is favored on sucrose‐rich substrates (Adesulu‐Dahunsi et al. 2018; Di Cagno et al. 2006). Screening candidate starters for this trait is therefore a relevant preliminary step when the target matrix is a fruit purée.

A second trait of technological interest is the ability of LAB to limit mold growth. Fruit‐based matrices are particularly susceptible to fungal spoilage, and biological control through fermentation is an alternative to chemical preservatives (Dalié et al. 2010). The effect is generally attributed to the combined action of organic acids and of the associated pH decrease, together with low‐molecular‐weight compounds such as antifungal peptides and phenolic derivatives (Muhialdin et al. 2018; Zapaśnik et al. 2022). This activity varies considerably between species and strains and cannot be predicted from taxonomic assignment alone (Souza et al. 2023), which makes its experimental evaluation necessary when selecting starter cultures.

Despite the technological relevance of these traits, research on banana fermentation has concentrated on by‐products, and in particular on peel, used for bioenergy production or as a source of extractable bioactive compounds (Hashim 2023; Mohd Zaini et al. 2022; Muhammad Ansari et al. 2023). The pulp has been fermented far less often, and few studies have compared different LAB species in this matrix; to our knowledge, none has done so using untargeted metabolomics. How single strains and consortia reshape the metabolite profile of banana pulp is therefore still largely unknown.

We characterized the controlled lactic fermentation of banana pulp by *L. plantarum* UC9052, *L. brevis* UC9051 and *W. cibaria* UC9053, applied as single and mixed cultures. The strains were first characterized in vitro for EPS production, antifungal activity, and carbohydrate metabolism. Fermentation was then followed through viable counts and pH; the fermented purée was freeze‐dried, and the metabolite profile of the resulting matrix was characterized by untargeted UHPLC‐HRMS, with the aim of evaluating it as a potential functional ingredient. This preliminary study characterized the fermented and freeze‐dried matrix on its own, while its behavior once incorporated into a food formulation, where composition and processing may change its properties, remains to be assessed.

## 2 Materials and Methods

### 2.1 Technological Strain Characterization

#### 2.1.1 Bacterial Strains

Three LAB strains were used in this study: *L. brevis* UC9051, *L. plantarum* UC9052, and *W. cibaria* UC9053. The strains are maintained in the internal culture collection of the Department of Sustainable Food Process, Università Cattolica del Sacro Cuore (Cremona, Italy), from which they are available on request. They were isolated from plant‐based samples and have not been deposited in a public culture collection. Lyophilized cultures were reactivated by cultivation in MRS broth (Oxoid, Italy) for 48 h at 37°C under anaerobic conditions. Then, they were streaked in MRS agar, and a colony was resuspended in MRS broth to be used for the following tests. The strains were characterized for their ability to produce exopolysaccharides (EPS), antifungal activity and sugar metabolism, as described in the following sections.

#### 2.1.2 EPS Production

Detection of the ability to synthesize EPS was done as previously described (Di Cagno et al. 2006). The three strains were subcultured in MRS broth (pH 5.6) for 24 h at 37°C under anaerobic incubation. The day after, samples were vortexed and 5 μL of the sample were spotted on MRS agar plates enriched with 146 mM of glucose and 292 mM of sucrose. Plates were then maintained at 37°C for 6 days under anaerobic incubation. The ability to produce EPS was determined by measuring the mucous zone around the spot onto the plate.

#### 2.1.3 Antifungal Activity

The protocol followed for the determination of antifungal activity was carried out as previously described (Dal Bello et al. 2007). The three strains were individually cultured in MRS broth and maintained at 37°C under anaerobic incubation. After 24 h, 10 μL were spotted in triplicate on MRS agar plates and maintained under anaerobic incubation for 48 h at 37°C. A suspension of *Aspergillus cibarius* (Università Cattolica culture collection) spores was collected from colonies grown on Rose Bengal agar (Oxoid Italia) by homogenizing them for 2 min in saline solution containing 0.05% Tween 20. After filtration, spores were counted in a Thomas chamber and the suspension was adjusted to 10^4^ spores/mL; the final concentration was confirmed by plate count on Rose Bengal agar (Oxoid). Subsequently, Malt Extract Soft Agar (Oxoid Italia) was inoculated with the standardized spore suspension of 1 mL of spore suspension per 10 mL of soft medium (0.8% agar). Ten mL of the inoculated medium were then poured onto the MRS plates containing the grown bacterial colonies. Plates were incubated at 25°C for 5 days. Antifungal activity was determined by the appearance of a clear inhibition halo in the overlay agar around bacterial spot. Two controls were included in each assay: sterile MRS broth (10 μL) spotted onto separate plates to verify that the medium itself did not inhibit mold growth, and uninoculated MRS agar plates overlaid with the spore suspension and incubated under the same conditions to confirm normal development of the mold in the absence of LAB.

#### 2.1.4 Strain Metabolism Characterization

Carbohydrate metabolism was analyzed using the API 50 CH system (bioMérieux Italia). LAB strains were grown in MRS broth at 37°C for 24 h under anaerobic incubation, washed three times with saline solution (0.9% NaCl), and resuspended to a turbidity corresponding to McFarland standard 2. The suspension was transferred into API 50 broth medium and distributed into each microtube of the API gallery, with the first well inoculated with medium alone as a negative control. The microtubes were sealed with sterile paraffin oil to ensure anaerobic conditions and prevent evaporation. Finally, strips were incubated at 37°C, and the color change was monitored at 24, 48, and 72 h following the manufacturer instructions.

### 2.2 Lab‐Scale Fermentation of Banana Purée

Bananas at ripening stage C4 (“more yellow than green”), defined by the ripening classification reported by Gomes and colleagues (Gomes et al. 2013), were selected from a retail store. In a sterile environment, the pulp was peeled, blended (G3 Ferrari, G20155 Realmix 2) for 1 min at maximum power, and diluted 1:1 with sterile distilled water. The purée was divided into 150 mL aliquots. A control tank containing only banana purée was prepared, while additional tanks were inoculated with LAB, either individually or in blends as described in Table 1. Since the substrate was not thermally treated, the control tank underwent spontaneous fermentation by the native microbiota. Cultures grown in MRS broth were harvested by centrifugation, washed in saline solution (0.9% NaCl), and resuspended to a turbidity corresponding to McFarland standard 1. The suspensions were then diluted in the purée to reach a final inoculum of approximately 6 log_10_ CFU/g, and the actual inoculum level was verified by plate count at T0. Fermentation was carried out in an Orbital Shaker Incubator ES‐20 (Grant‐bio) at 37°C and 150 rpm for 48 h, in triplicates. Agitation was applied because the diluted purée remained a dense and non‐Newtonian matrix, in which the inoculum would otherwise distribute unevenly and solids would tend to sediment. Continuous mixing was therefore used to keep the matrix homogeneous throughout fermentation and to ensure that the samples withdrawn at each time point were representative of the whole tank.

**TABLE 1:** Sample tested for the fermentation of banana purée.

Sample	Strains
N	Control without inoculum
B	*L. brevis* UC9051
P	*L. plantarum* UC9052
W	*W. cibaria* UC9053
PB	*L. plantarum* UC9052*+ L. brevis* UC9051
BW	*L. brevis* UC9051*+ W. cibaria* UC9053
PW	*L. plantarum* UC9052*+ W. cibaria*
PBW	*L. plantarum* UC9052*+ L. brevis* UC9051*+ W. cibaria* UC9053

### 2.3 Fermented Product Characterization

#### 2.3.1 Determination of pH and Microbial Count

Fermented banana purée samples were analyzed at three time points, immediately after the inoculation, after 24 h (T24), and after 48 h (T48). At each sampling point, 1 mL of fermented sample was collected from the fermentation tanks and subjected to serial dilutions. Samples were plated on MRS agar to enumerate LAB, whereas potential contaminants such as *Enterobacteriaceae*, *Bacillus cereus* , and yeasts and molds were quantified on Violet Red Bile Glucose Agar (Oxoid Italia), Polymyxin Egg Yolk Mannitol Bromothymol Blue Agar (Oxoid Italia), and Rose Bengal Chloramphenicol Agar Base (Oxoid Italia), respectively. The pH of the fermented banana purée was measured using a calibrated pH meter (Hanna edge blue‐HANNA instruments‐AR‐IT). Measurements were performed on aliquots collected from the fermentation tanks at each time point previously reported. Viable cell counts were expressed as log_10_ CFU/mL. Standard deviations were calculated after log transformation to account for the exponential nature of microbial growth.

#### 2.3.2 Lyophilization of Fermented Banana Purée

Fermented samples were subjected to lyophilization to improve the shelf life and last longer without being affected by microbiological alteration and maintain the nutritional characteristics. Each fermented sample was lyophilized individually using a lyophilizer (Buchi LyoVapor L‐200‐BUCHI Italia s.r.l) and the following parameters: −55°C for 48 h. Only the samples collected after 24 h of fermentation (T24) were freeze‐dried, as bacterial growth curves indicated that cultures had reached the stationary phase between T24 and T48. Therefore, the 24 h time point was selected as the final fermentation stage, representing the maximum metabolic activity without significant further changes in cell growth or metabolite production.

#### 2.3.3 Screening of Bioactive Compounds Through Untargeted Metabolomics

Samples were prepared by mixing 1 g of lyophilized material with 10 mL of 80% methanol in separate falcon tubes. These latter were then vortexed and subjected to ultrasonic treatment in a sonication bath (DU‐32 ARGOLab, Milan, Italy) at the maximum power of 120 W for 5 min. Following sonication, the falcon tubes underwent centrifugation at 2800*g* for 5 min using a Thermo Scientific SL 40 FR centrifuge (Thermo Fisher Scientific, Osterode am Harz, Germany). Then, 2 mL of the supernatants were transferred to new Eppendorf tubes and centrifuged at 15000*g* for 2 min, after which the supernatant was stored at −18°C overnight. The following day, the supernatant was filtered through a 0.22 μm filter into ultra‐high‐performance liquid chromatography (UHPLC) vials and stored at −18°C for subsequent analysis.

Bioactive compounds were then screened using UHPLC coupled with high‐resolution mass spectrometry (HRMS), based on an Orbitrap Q‐Exactive Focus (Thermo‐Scientific) with a nominal resolution of 70,000 FWHM at 200 m/z. The mobile phase consisted of water and acetonitrile, with a gradient chromatography (from 6% to 94% acetonitrile) over 35 min, and 0.1% formic acid was added as a phase modifier. Chromatographic separation was carried out using an Agilent Zorbax Eclipse Plus column, with a 6 μL injection volume for each sample. The HRMS parameters were optimized based on the work of Rocchetti and O'Callaghan (2021). Raw data from the analysis were processed using the MS‐DIAL software (version 4.90) (Tsugawa et al. 2015). The annotated mass features were aligned through an automatic peak search following LOWESS normalization against pooled Quality Control samples (i.e., containing small aliquots of each sample extract). Compound annotation was performed according to a Level 2 of confidence of annotation (i.e., putatively annotated compounds with MSMS confirmation) by comparing the obtained spectra against reference databases, including FooDB (https://www.foodb.ca) and the Mass Bank of North America. The criteria for compound identification and classification included a mass range of 80–1200 m/z, with a minimum peak height of 10,000 cps. Peak centralization for both MS and MS/MS was conducted with tolerances of 0.01 Da and 0.05 Da, respectively.

In addition to qualitative annotation, a semi‐quantitative evaluation was carried out for selected chemical classes using external calibration curves, as previously reported (Bozzetti et al. 2025; Rocchetti et al. 2024). Particularly, all the compounds identified were grouped into different typical subclasses and then semi‐quantified according to hydroalcoholic standard solutions of pure standard compounds analyzed under the same instrumental conditions. Semi‐quantification was therefore expressed as equivalents of the reference standards available for each class, namely cyanidin equivalents for anthocyanins, catechin equivalents for flavan‐3‐ols, luteolin equivalents for flavones and other flavonoids, quercetin equivalents for flavonols, 5‐methyltetrahydrofolate (5‐MTHF) equivalents for folate metabolites, folic acid based on its specific calibration curve, oleuropein equivalents for other polyphenols, ferulic acid equivalents for phenolic acids, resveratrol equivalents for stilbenes, and β‐carotene equivalents for terpenoids. In this regard, a linear fitting (*R* ^2^ > 0.98) was built and used for semi‐quantification, expressing the results as μg of equivalents (Eq.)/100 g. Metabolomic analyses were performed on two of the three biological replicates of each fermentation, each injected twice, giving four analytical observations per sample (*n* = 4).

### 2.4 Statistical Analysis

Microbiological analyses and pH measurements were carried out in triplicate, while metabolomic analyses were based on two biological replicates injected twice (*n* = 4); in the multivariate analyses, the two analytical replicates of each biological replicate were treated as independent observations. Results are expressed as mean ± standard deviation. Microbial counts were log_10_ transformed prior to statistical analysis to account for the exponential nature of microbial growth. Descriptive statistics and statistical analyses were performed using Microsoft Excel (Microsoft Corp., Redmond, WA, USA). Differences among samples and fermentation times were evaluated by one‐way analysis of variance (ANOVA), and statistical significance was established at *p* < 0.05. When the ANOVA indicated significant differences, means were separated by Tukey's HSD post hoc test (*p* < 0.05). For untargeted metabolomics data obtained by UHPLC‐HRMS, raw data processing, peak alignment, and normalization were performed using MS‐DIAL software, while multivariate statistical analyses, including principal component analysis (PCA), hierarchical clustering analysis (HCA), and partial‐least‐squares discriminant analysis (PLS‐DA), were carried out using MetaboAnalyst 6.0 to explore differences in metabolic profiles among fermented samples. Also, SIMCA 16.0 (Umetrics, Malmo, Sweden) was used to perform supervised OPLS‐DA prediction model, after cross‐validation by ANOVA (*p* < 0.01) and overfitting exclusion by permutation testing (*n* = 200).

## 3 Results and Discussion

This study aimed to characterize the technological, microbiological, and metabolic behavior of selected LAB during the fermentation of banana purée, using both single and mixed cultures of the three strains. The choice of banana as a fermentation substrate was motivated by its wide availability, favorable chemical composition, and documented suitability for lactic fermentation, as previously reported for banana purée under different processing and ripening conditions (Gallo et al. 2021).

Beyond its technological aptitude, the fermentation of fruit matrices using LAB has attracted increasing interest as a sustainable strategy to improve microbial safety, extend shelf life, and enhance the functional properties of fruit‐based products, while simultaneously contributing to the reduction of food losses along the supply chain (Chiarini et al. 2024; Mia et al. 2025). In this context, bananas at intermediate ripening stages, although generally suitable for fresh consumption, may be excluded from retail distribution due to aesthetic defects or lack of uniformity, despite maintaining an appropriate nutritional and technological quality. Such fruits therefore represent a promising raw material for the development of fermented functional products through controlled lactic fermentation, offering an effective approach for their valorization and upcycling (Râpă et al. 2024). In this study, bananas at ripening stage C4 were selected as they represent a technological compromise between sufficient sugar availability derived from starch degradation and a still‐adequate structural integrity of the pulp, allowing for a controlled and reproducible fermentation process.

### 3.1 Technological Characterization of Bacterial Strains

Cultures of the three strains either alone or in combination were used to ferment banana purée. Before testing the LAB strains in the fermentation trials using banana purée as primary matrix, a technological and metabolic analysis was performed on the three strains object of the study.

#### 3.1.1 EPS Production

EPS synthesis by LAB is a technologically relevant trait in fruit‐based fermentation, since these biopolymers can increase product viscosity and water‐holding capacity (Guérin et al. 2020). With the aim of evaluating the technological potential of the selected LAB strains in improving the texture and stability of fermented banana products, their ability to produce EPS was individually assessed. The analysis, performed in triplicate, showed that only *W. cibaria* UC9053 was able to produce EPS with a 1.00 ± 0.30 cm of mucous zone around the spot, whereas *L. plantarum* UC9052 and *L. brevis* UC9051 did not show any detectable EPS activity under the tested conditions. The sugar composition of the pulp was not determined experimentally. Data reported for Cavendish bananas at a comparable ripening stage indicate a total starch content of about 57% and a free sugar content of about 183 mg glucose/g, values that reflect the progressive conversion of starch into fermentable sugars taking place at this stage (Huang et al. 2024).

This finding is consistent with previous studies reporting *W. cibaria* as an efficient EPS producer, particularly when cultivated on sucrose‐rich media that promote glycosyltransferase activity and polysaccharide synthesis (Adesulu‐Dahunsi et al. 2018). *W. cibaria* was the only strain producing a measurable EPS zone under the tested conditions, a trait potentially relevant for the viscosity and water‐holding capacity of fermented matrices.

In contrast, EPS production in *L. brevis* (Du et al. 2022) and *L. plantarum* (Zhou et al. 2016) has been shown to be highly strain‐dependent and influenced by environmental parameters such as pH, temperature, and the type and concentration of available carbon sources. Therefore, the absence of EPS formation observed here likely reflects suboptimal growth conditions for EPS biosynthesis rather than the complete lack of metabolic potential.

#### 3.1.2 Antifungal Activity

The ability of LAB to inhibit mold growth is an additional technological trait in fermented foods, since it can limit spoilage and reduce the need for chemical preservatives (Hui and Evranuz 2012). To assess the antifungal potential of the tested LAB strains and their possible contribution to improving the microbiological stability and shelf life of fermented banana products, the antifungal activity of the three strains was evaluated. The three strains were individually tested against *A. cibarius* using an agar spot inhibition assay. No inhibition was observed around the sterile MRS broth spots, and the mold developed uniformly on the uninoculated control plates. All strains exhibited clear antifungal activity, confirming their capability to suppress fungal growth under the tested conditions. The three strains showed respectively a mean inhibition halo of 1.93 ± 0.12, 2.23 ± 0.15 and 2.17 ± 0.31 cm, with no significant differences among the three strains (*p* = 0.257, one‐way ANOVA). These behaviors are in line with previous studies indicating that strains of *W. cibaria* (Souza et al. 2023), *L. plantarum* , and *L. brevis* (Dalié et al. 2010) were able to inhibit mold growth. The mitigation effect observed in this study can reasonably be attributed to the combined action of several metabolites typically produced by LAB, such as lactic and acetic acids, hydrogen peroxide, and phenolic compounds. These molecules are known to lower the pH and create conditions that are unfavorable for mold metabolism (Muhialdin et al. 2018). Although the contribution of other bioactive compounds, such as small peptides or proteinaceous substances, cannot be excluded, their role was not specifically investigated here.

#### 3.1.3 Sugar Metabolism

Fermentation kinetics and the production of organic acids can be influenced by the ability of LAB to metabolize different carbohydrates with the synthesis of metabolites with nutritional relevance and contributing, at the same time, to aroma characteristics. To evaluate the carbohydrates utilization profiles of the selected strains, an API 50 CHL test was performed after incubation at 37°C for 72 h. The metabolic patterns obtained for *L. plantarum* UC9052 (P), *L. brevis* UC9051 (B), and *W. cibaria* UC9053 (W) are summarized in Table 2. The three strains differed in their carbohydrate fermentation profiles. *L. plantarum* fermented the broadest range of substrates, including pentoses (L‐arabinose, D‐ribose), hexoses (glucose, fructose, galactose), disaccharides (maltose, sucrose, cellobiose), and phenolic glycosides (arbutin, salicin). These results aligned with previous studies highlighting the extensive carbohydrate adaptability of *L. plantarum* , supported by its flexible genomic organization and wide repertoire of phosphotransferase systems and glycosidases (Lu et al. 2018).

**TABLE 2:** Carbohydrate fermentation profiles determined by API Test 50 CHL. The tested strains were: P ( *L. plantarum* UC9052), B ( *L. brevis* UC9051), W ( *W. cibaria* UC9053). Sugar fermentation intensity is indicated as follows: ++ = strong positive reaction, + = positive reaction, − = no reaction.

Sugar	Lab strains		
	P	B	W
L‐arabinose	++	−	+
D‐ribose	+	++	+
D‐xylose	−	++	+
D‐galactose	++	−	++
D‐glucose	++	+	++
D‐fructose	++	+	++
D‐mannose	++	−	++
D‐mannitol	++	++	−
D‐sorbitol	++	−	−
Methyl alpha‐D‐glucopyranoside	++	−	−
Methyl alpha‐D‐mannopyranoside	++	−	−
N‐acetylglucosamine	++	+	+
Amygdalin	++	−	+
Arbutin	++	−	+
Salicin	++	−	+
D‐cellobiose	++	−	++
D‐maltose	++	+	++
D‐lactose (bovine)	++	−	−
D‐melibiose	++	+	−
D‐sucrose	++	−	−
D‐trehalose	++	−	−
D‐melezitose	++	−	−
D‐raffinose	++	−	−
Gentibiose	+	−	+
D‐turanose	++	−	−
D‐arabitol	−	+	−
Potassium gluconate	+	+	+

In contrast, *L. brevis* exhibited a narrower metabolic profile, showing positive fermentation primarily for pentoses and polyols (D‐xylose, D‐ribose, D‐mannitol), aligning with its ecological adaptation to plant niches where pentoses and sugar alcohols are predominant, and where metabolic efficiency rather than diversity provides a competitive advantage (Hacıoglu and Kunduhoglu 2021).

*W. cibaria* showed an intermediate phenotype, efficiently fermenting simple sugars and disaccharides such as glucose, fructose, galactose, and maltose while showing no activity on polyols. This pattern reflects its typical heterofermentative metabolism and its known role in vegetable and cereal fermentations, where hexoses are converted into lactic acid, acetic acid, and CO_2_, a pathway that contributes to both flavor development and pH modulation during fermentation (Fusco et al. 2015).

The ability of *L. plantarum* to metabolize glucose, fructose, and sucrose, and of *W. cibaria* to metabolize glucose and fructose, the main sugars naturally present in banana pulp (Lizana 1976), suggests their suitability for this fruit. Their metabolic activity is expected to promote rapid acidification and stable fermentation dynamics, whereas *L. brevis* , although less versatile, may contribute to flavor diversification through the production of secondary metabolites derived from pentose and polyol metabolism.

### 3.2 Characterization of Banana Purée Fermented With Different LAB Combinations

#### 3.2.1 Microbial Growth and pH During Fermentation

Bacterial growth was monitored to follow microbial adaptation and metabolic activity within the fruit matrix. In LAB‐driven fermentations, cell proliferation is typically associated with the production of organic acids and other primary metabolites that shape the physicochemical and sensory characteristics of the product (Leroy and De Vuyst 2004).

As shown in Table 3, viable LAB counts on MRS medium increased between T0 and T24 in all inoculated samples, indicating adaptation to the banana substrate. Although all cultures were standardized to a nominal inoculum of approximately 6 log_10_ CFU/mL, viable counts at T0 differed by up to about one log unit among strains. Standardization was based on turbidity (McFarland standard 1), which does not correspond to the same number of colony‐forming units across species, as cell size, cell shape, and the tendency to form chains or aggregates all affect the relationship between optical density and viable counts. These initial differences were leveled out within 24 h, when all inoculated samples reached 7.9–9.1 log_10_ CFU/mL, the mixed cultures growing as well as or better than the corresponding single strains. Counts then remained stable between 24 and 48 h in all inoculated samples (*p* > 0.05), indicating a transition towards the stationary phase, with the highest final densities recorded for P, PB, and PBW. The spontaneously fermented control remained 4–5 log units below the inoculated samples throughout, although its counts increased significantly over time (*p* < 0.01).

**TABLE 3:** Viable counts of LAB (log_10_ CFU/mL) and pH values in banana purée fermented with single and mixed LAB cultures for 48 h.

Sample	LAB (log_10_ CFU/mL)			pH		
	T0	T24	T48	T0	T24	T48
P	5.95 ± 0.15 b	9.11 ± 0.05 a	9.10 ± 0.32 a	4.86 ± 0.02 a	3.43 ± 0.05 c	3.27 ± 0.02 c
B	5.09 ± 0.21 d	7.90 ± 0.61 b	8.51 ± 0.09 b	4.96 ± 0.02 a	4.45 ± 0.28 a	3.80 ± 0.10 b
W	6.42 ± 0.08 a	8.74 ± 0.00 a	8.27 ± 0.30 b	4.93 ± 0.04 a	4.53 ± 0.05 a	3.91 ± 0.30 b
PB	5.55 ± 0.18 c	9.13 ± 0.08 a	9.40 ± 0.22 a	4.97 ± 0.04 a	3.52 ± 0.03 c	3.29 ± 0.01 c
PW	6.21 ± 0.15 a	8.79 ± 0.12 a	8.72 ± 0.11 b	4.89 ± 0.03 a	3.60 ± 0.02 c	3.36 ± 0.01 c
BW	6.30 ± 0.05 a	8.10 ± 0.54 b	8.39 ± 0.34 b	4.86 ± 0.05 a	4.32 ± 0.07 b	3.71 ± 0.15 b
PBW	6.11 ± 0.09 a	8.87 ± 0.14 a	8.85 ± 0.10 a	4.74 ± 0.09 b	3.57 ± 0.06 c	3.36 ± 0.03 c
N	< LOD	3.54 ± 0.23 c	4.73 ± 0.14 c	4.91 ± 0.05 a	4.64 ± 0.07 a	4.40 ± 0.12 a

*Note:* Values are means ± standard deviation of three biological replicates, calculated on log_10_‐transformed data. Different letters within the same column indicate significant differences among samples (one‐way ANOVA followed by Tukey's HSD test, *p* < 0.05); no letters are given where the ANOVA was not significant.

Abbreviations: B, *L. brevis* UC9051; N, spontaneously fermented control; *P*, *L. plantarum* UC9052; PB, PW, BW and PBW, mixed cultures; W, *W. cibaria* UC9053.

In addition to fermentation performance, selected microbial groups of hygienic interest were evaluated to assess their evolution during the process. This aspect is relevant in fruit‐based matrices that are not subjected to thermal pre‐treatment, where native microbiota and initial contamination may significantly influence fermentation dynamics, microbial competition, and product quality. *B. cereus* , yeasts and molds remained below the detection limit in all samples at every sampling time (data not shown), indicating that the process conditions did not support their development. *Enterobacteriaceae* were detected at T0 in all samples in 1.3–3.4 log_10_ CFU/mL (*p* = 0.112), reflecting the contamination of the initial fruit matrix. After 24 h of fermentation, they were below the detection limit (< 1.00 log_10_ CFU/mL) in all LAB‐inoculated samples except in B (7.71 ± 0.87 log_10_ CFU/mL) and in W (1.94 ± 0.96 log_10_ CFU/mL), while the spontaneously fermented control (N) increased to 5.71 ± 0.02 log_10_ CFU/mL. Notably, B, W, and N samples were the only ones in which *Enterobacteriaceae* remained detectable at 24 h, corresponding to the highest pH values (> 4.4), whereas all the other fermentations reached lower pH levels (≤ 4.3). By 48 h, *Enterobacteriaceae* counts were below the detection limit in all samples, including B, W, and control (N) samples, this last also showing a further decrease in pH to values below 4.4. The pH drop observed in the control is consistent with acidification driven by spontaneous fermentation by the native LAB microbiota, which likely contributed to conditions unfavorable for *Enterobacteriaceae* survival (Zapaśnik et al. 2022).

pH was monitored as an indicator of microbial metabolic activity, reflecting the conversion of fermentable sugars into organic acids. In LAB‐driven fermentations, acidification represents a key technological parameter, as it contributes to product safety, texture stabilization, and flavor development (Leroy and De Vuyst 2004). Over the 48‐h fermentation period, the pH exhibited a clear decreasing trend in nearly all samples, consistent with active fermentative metabolism. Two distinct patterns were observed. In samples inoculated with *L. plantarum* , alone (P) or in combination (PB, PW, PBW), the initial pH of 4.7–5.0 dropped sharply within the first 24 h, reaching values of 3.4–3.6, and then remained essentially unchanged, whereas in B, W and BW the decrease was more gradual and continued throughout the process (Table 3). This pronounced acidification reflects the production of lactic and acetic acids, typical metabolites of LAB responsible for the rapid decrease in pH during the exponential growth phase. Between 24 and 48 h, a further progressive decrease (*p* < 0.05) was observed in samples fermented with *L. brevis* UC9051 (B), *W. cibaria* UC9053 (W), and their combined culture (BW), whereas no significant variation was detected in the other formulations, which had already reached pH values below 3.6 at 24 h.

The different acidification patterns observed among the tested strains can be attributed to species‐specific physiological traits. LAB display species‐dependent differences in acid tolerance and organic acid production, reflecting their distinct metabolic regulation. *L. plantarum* typically exhibits a faster and more pronounced acidification, supported by its high acid resistance and efficient carbohydrate metabolism (Siezen and van Hylckama Vlieg 2011). In contrast, *L. brevis* and *W. cibaria* show a more gradual pH decrease, likely due to lower acidogenic capacity and an earlier growth arrest under acidic conditions (Yu et al. 2020). This trend is consistent with the microbial counts, which reached a plateau between T24 and T48, confirming the stabilization of bacterial growth and metabolic activity. The control sample (N) exhibited a slight but statistically significant decrease in pH over the incubation period (from 4.91 ± 0.05 to 4.40 ± 0.12; *p* < 0.05), consistent with limited metabolic activity by the native microbiota naturally present in the banana pulp. However, the extent of acidification was noticeably lower than in the inoculated samples, indicating that the marked pH drop observed in the latter was predominantly driven by added starter LAB rather than by the indigenous microbiota. The drop in pH during the first 24 h is relevant for product stability, since it can limit the growth of spoilage organisms in the banana matrix (Leroy and De Vuyst 2004). The subsequent stabilization of pH suggests that extending fermentation beyond this stage offers little additional benefit, since acid accumulation may begin to limit bacterial viability and metabolic activity.

Finally, the appearance and odor of the fermented samples were assessed qualitatively during sampling. After 24 h the purée was slightly more fluid than at the start and had turned light brown, with a sweet odor reminiscent of cooked banana. Between 24 and 48 h the color darkened further and the odor became slightly sharper. These are informal observations made by the operators, without a trained panel or standardized scales; taste was not assessed.

#### 3.2.2 The Effect of Lyophilization on LAB Viability

Based on the results described above, T24 was selected as the endpoint for product formulation, corresponding to the phase of maximal LAB viability and metabolic activity prior to the stationary phase. Indeed, at this time point all inoculated samples reached high LAB counts and exhibited active growth dynamics. *Enterobacteriaceae* were below the detection limit in all inoculated samples except B and W (Section 3.2.1), consistent with their slower acidification. Samples were therefore freeze‐dried after 24 h of fermentation to preserve the viability and functional traits of the lactic acid bacteria. The production of a freeze‐dried matrix is particularly relevant for the development of functional ingredients, as it enables improved shelf‐life, easier handling, and the incorporation of the fermented product into different food formulations.

As shown in Figure 1, all fermented samples exhibited a significant reduction in LAB viable cell counts after freeze‐drying, with the magnitude of the loss depending on the microbial strain or consortium. In P sample, *L. plantarum* UC9052 counts decreased significantly from 9.11 ± 0.05 to 7.80 ± 0.09 log_10_ CFU/mL (≈1.3 log reduction). In the B sample, *L. brevis* UC9051 showed the lowest post‐drying survival, decreasing from 8.20 ± 0.58 to 6.93 ± 0.07 log_10_ CFU/mL. In contrast, in the sample fermented with *W. cibaria* UC9053, this strain retained 7.91 ± 0.07 log_10_ CFU/mL compared to 8.74 ± 0.11 in the fresh state (≈0.8 log reduction). A significant reduction of viable LAB counts compared to the initial one was observed also among the samples fermented with mixed cultures. Bacterial loads between 7.60 ± 0.07 and 7.87 ± 0.06 log_10_ CFU/mL were detected after lyophilization, starting from initial values between 8.33 and 9.14 log_10_ CFU/mL. Taken together, these formulations exhibited reductions of approximately 1.0–1.5 log units. Within the observed variability, survival after freeze‐drying was comparable between single‐strain and multi‐strain fermentations (Figure 1). Therefore, based on the present data, mixed cultures did not show a clear advantage over single strains in terms of freeze‐drying tolerance. The uninoculated control (N) also showed measurable growth on MRS medium, with counts reaching 3.58 ± 0.23 log_10_ CFU/mL before freeze‐drying and decreasing to 2.07 ± 0.06 log_10_ CFU/mL afterwards (≈1.5 log units). These microorganisms likely originated from the native microbiota of the banana pulp and/or from environmental contaminants, as the substrate was not sterilized prior to fermentation. The log reduction observed in the control was therefore consistent with that recorded in the inoculated samples, indicating that freeze‐drying imposed a similar stress on both indigenous and added microbiota. These results are in line with evidence showing that LAB can rapidly achieve high cell concentrations (≥ 8.00 log_10_ CFU/mL) in fruit‐based matrices within 24 h, thereby contributing to the safety and functional quality of the product (Garcia et al. 2020). In our study, the attainment of around 9.00 log_10_ CFU/mL by *L. plantarum* UC9052 and mixed cultures within 24 h, together with the decline of *Enterobacteriaceae* below the detection limit by 48 h in all samples, indicates that the fermentation conditions became progressively unfavorable to this group of contaminants. Moreover, the maintenance of viable cells after freeze‐drying (mean 7.60–7.91 log_10_ CFU/mL depending on the strain), consistent with previous studies on the survival of LAB in the presence of lyoprotective excipients (Cui, Hu, et al. 2022; Cui, Pan, et al. 2022), supports the feasibility of developing freeze‐dried fruit‐based fermented products retaining viable LAB, although their stability during storage was not evaluated in this study.

**FIGURE 1:**
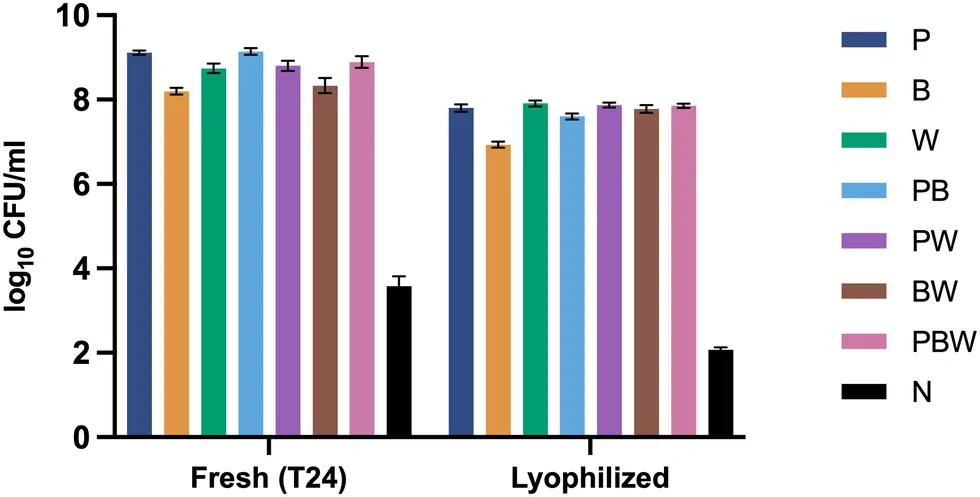
Total LAB counts in fermented banana purée after 24 h (fresh T24) and in fermented banana purée after lyophilization treatment (lyophilized). Cell counts are expressed in log_10_ CFU/mL.

#### 3.2.3 Effects of Fermentation on the Qualitative and Semi‐Quantitative Metabolomic Profiles

Untargeted metabolomics was used to characterize the biochemical changes occurring during fermentation and their impact on the composition of the final matrix. In fruit‐based fermentations, LAB modify metabolite composition through enzymatic activities that release, convert, or synthesize bioactive compounds, including phenolics, vitamins, and amino acid derivatives (Yang et al. 2024).

The UHPLC‐HRMS untargeted metabolomic analysis performed on fermented and lyophilized banana purée enabled the putative annotation of 433 metabolites across the analyzed samples (Table S1). Under the adopted experimental conditions, a broad diversity of chemical entities was detected, with amino acids, peptides, and analogues representing the predominant class (20%), followed by carbohydrates and carbohydrate conjugates (8.3%), together with several phenolic subclasses, including phenolic acids and flavonoids (Figure 2). The untargeted approach was employed to investigate how fermentation with different LAB strains, namely *L. plantarum* UC9052 (P), *L. brevis* UC9051 (B), and *W. cibaria* UC9053 (W), applied as single cultures (P, B, W), binary consortia (PB, PW, BW), and a ternary consortium (PBW), modulated the metabolomic profile of banana purée compared with spontaneous fermentation (N).

**FIGURE 2:**
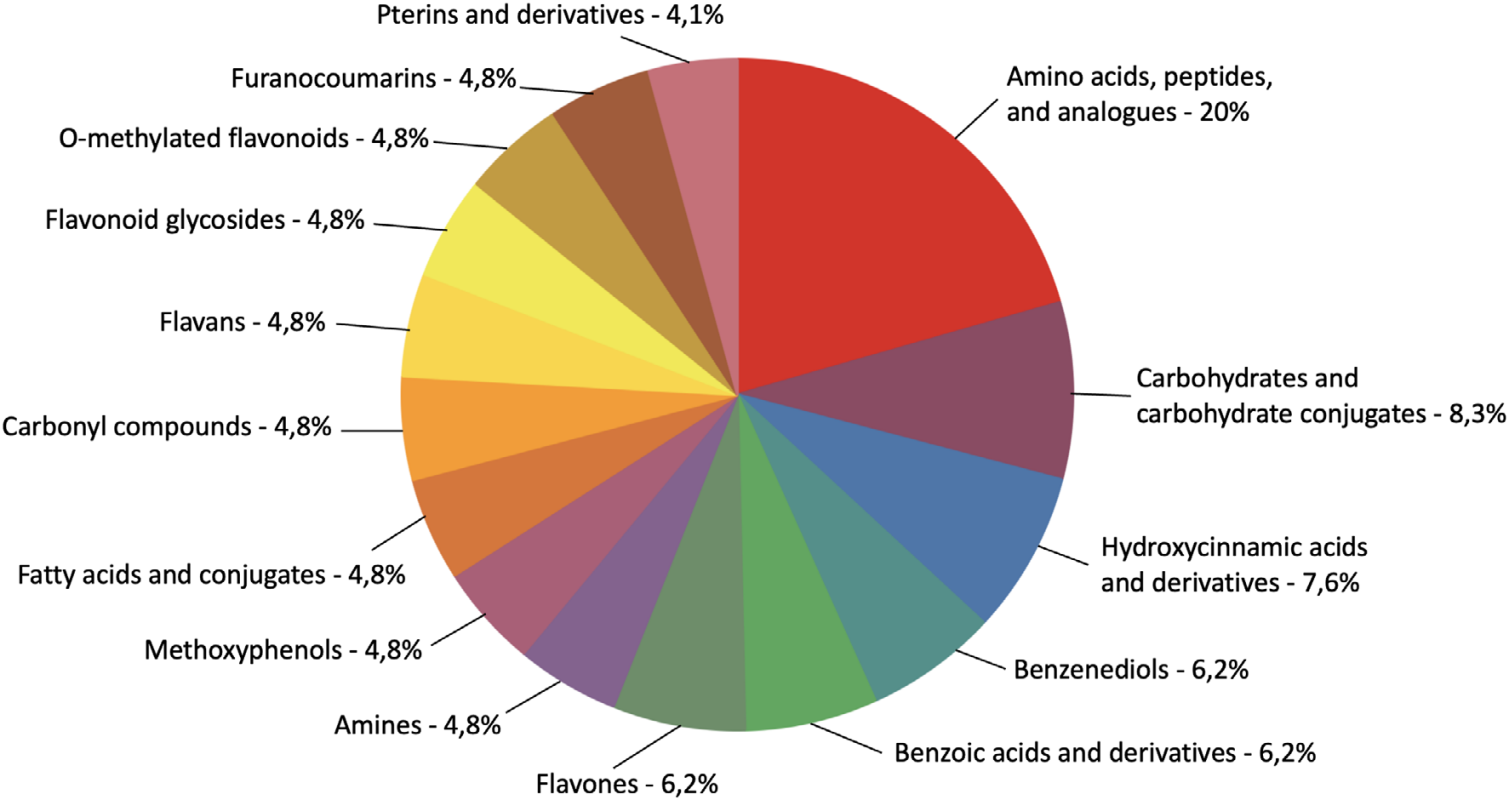
Pie chart showing the chemical classes annotated by untargeted metabolomics in the different samples considering the classes of metabolites containing at least two entries.

Unsupervised principal component analysis (PCA) revealed a separation between fermented samples and the control (Figure 3a). In particular, N was distinctly separated along PC1 (18.6% explained variance), whereas W displayed an intermediate positioning between the control and the remaining fermented samples. Treatments involving *L. plantarum* and *L. brevis* , either alone or in mixed cultures, formed more compact clusters, indicating closer metabolomic profiles. These trends were further confirmed by hierarchical heat map clustering analysis (Figure 3b), which identified three main groups: (i) N together with W, (ii) P, B, and PB, and (iii) PW, BW, and PBW. These results indicate that mixed fermentations produced a larger shift in the banana metabolome relative to control than single‐strain inoculations, particularly in the presence of *W. cibaria* . The apparent contrast between the position of W, which clustered with the control, and the larger shifts recorded in the consortia containing *W. cibaria* may be related to the metabolic traits of this species. *W. cibaria* UC9053 acidified the matrix slowly (pH 3.91 ± 0.30 at 48 h; Table 3) and, as a single culture, may therefore have had a limited capacity to modify the matrix within the fermentation time. In mixed cultures, where acidification was driven by *L. plantarum* or *L. brevis* , two additional activities of *W. cibaria* may become relevant. First, this was the only strain producing EPS under the conditions tested (Section 3.1.1), a trait associated with extracellular glucansucrase activity, which hydrolyzes sucrose to build the glucan chain while concomitantly releasing fructose (Besrour‐Aouam et al. 2021); as the strain fermented fructose but not sucrose (Table 2), this reaction would make an additional fermentable substrate available to the consortium. Second, its obligately heterofermentative metabolism yields acetate, ethanol and CO_2_ alongside lactate (Fusco et al. 2015), broadening the range of end products formed. These mechanisms were not investigated directly in the present study and are proposed as hypotheses consistent with the observed data.

**FIGURE 3:**
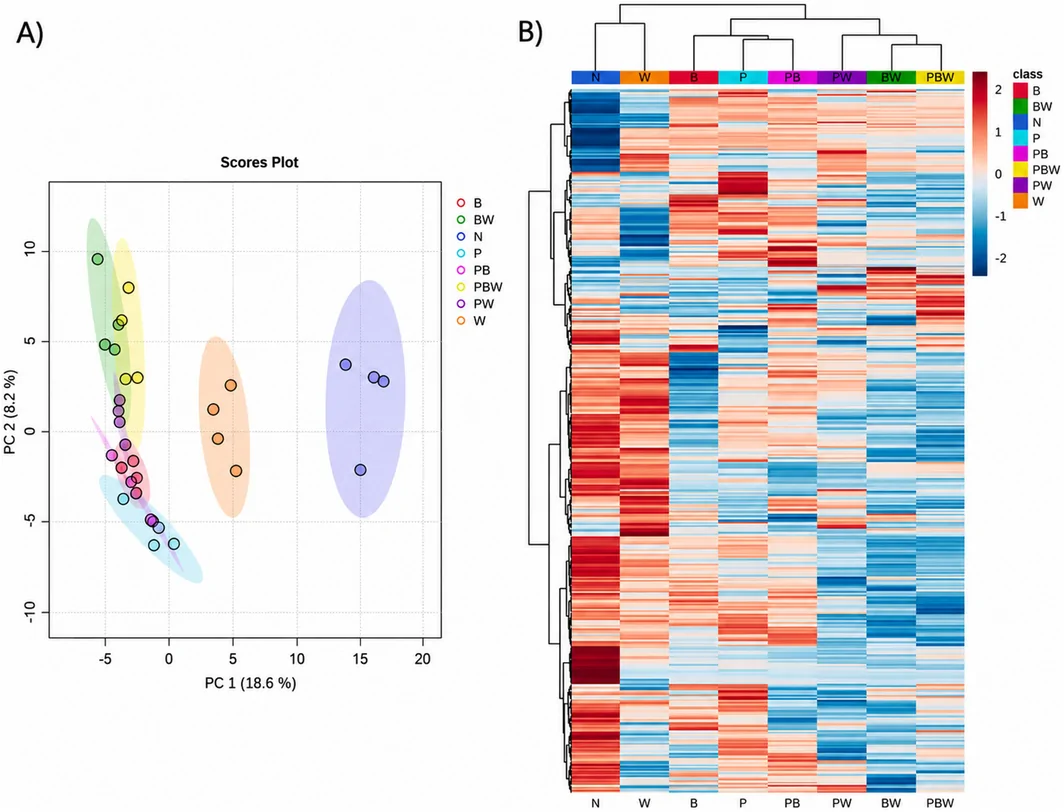
(A) Principal component analysis (PCA) of the main metabolites found in samples fermented with different starter strains. (B) Clustering heatmap; the normalized Log_10_ abundance value of each metabolite found in the fermented purée was considered.

A semi‐quantitative analysis (Table S1) based on raw abundance values highlighted significant differences among several metabolite classes, including flavanols, flavones, flavonols, folates, folic acid, phenolic acids, stilbenes, and other phenolics (ANOVA followed by Duncan's multiple range test in MetaboAnalyst 6.0, *p* < 0.05; post hoc groupings reported in Table S1). All concentrations reported below are semi‐quantitative, expressed as μg of equivalents of the reference standard selected for each chemical class, and are therefore suitable for comparisons within the present dataset rather than as absolute contents. Flavanol‐related metabolites generally decreased after fermentation, except in PBW, where higher levels were detected (i.e., 105.2 μg Eq./100 g). This behavior may reflect active microbial catabolism of flavan‐3‐ols and their conversion into lower molecular weight derivatives. Conversely, flavones increased in all fermented samples except B and P, with PW exhibiting the highest content (1085.2 μg Eq./100 g). Flavonols displayed a more heterogeneous trend, decreasing in single‐strain fermentations and BW, while remaining closer to control values in PB, PW, and PBW (Table S1). Regarding folate metabolism, total folate levels generally decreased following fermentation, with PB representing the only treatment maintaining levels comparable to the control (Table S1). Folic acid was detected exclusively in fermented samples, reaching a maximum semi‐quantitative value of 4.8 μg Eq./100 g in P, whereas it was not detected in N. This finding may reflect microbial folate biosynthesis and/or interconversion phenomena among reduced folate vitamers occurring during fermentation and sample processing (Czarnowska‐Kujawska and Paszczyk 2021). *L. plantarum* is among the LAB species most frequently associated with folate biosynthesis in plant matrices, although the net effect on total folate content is strain‐ and substrate‐dependent and both increases and decreases have been reported (Tan et al. 2025; Thompson et al. 2020). The levels of folate‐related metabolites detected here, however, should not be interpreted as a nutritionally relevant enrichment of the matrix. Folate levels were not increased by fermentation: the highest value was recorded in PB (106.6 μg Eq./100 g), essentially equal to that of the spontaneously fermented control (106.3 μg Eq./100 g), while all other treatments showed lower values. Even if these semi‐quantitative values were taken at face value, and disregarding the fact that they are expressed as equivalents of a reference standard rather than as dietary folate equivalents, the folic acid detected in fermented samples (up to 4.8 μg Eq./100 g in P) would correspond to approximately 1.5% of the population reference intake of 330 μg DFE/day established for adults (EFSA 2014). As the ingredient is expected to be incorporated at low inclusion levels into composite foods, the actual contribution per serving would be considerably lower. Ascorbic acid and 3‐indolelactic acid were assessed only as relative abundances and were not semi‐quantified, so no intake estimate can be derived for them, while flavonoids are not essential nutrients and no reference intake values exist for them. The interest of the present findings therefore lies in the strain‐dependent modulation of the metabolic profile of banana pulp, and in the persistence of compounds reported in the literature to be biologically active, rather than in a measurable contribution to recommended intakes (Ferraz et al. 2020; Kumar et al. 2025).

To further investigate treatment‐specific metabolic shifts, a supervised OPLS‐DA model was developed, showing a discrimination among N, W, and the remaining fermentation trials (Table 4). The model showed goodness‐of‐fit and predictive parameters of *R* ^2^ *X*(cum) = 0.613, *R* ^2^ *Y*(cum) = 0.97, and *Q* ^2^(cum) = 0.648, with model reliability further supported by permutation testing (*n* = 200). Given the number of biological replicates available, these parameters are reported as descriptive of the present dataset rather than as validated predictive performance. Variable importance in projection (VIP) analysis identified 142 discriminant metabolites, including spermine (VIP score = 1.11), thiamine (VIP score = 2.02), ascorbic acid (VIP score = 1.04), 5‐methyltetrahydrofolic acid (VIP score = 1.46), 3‐indolelactic acid (VIP score = 1.84), and 5‐(3′,5′‐dihydroxyphenyl)‐γ‐valerolactone 3‐*O*‐glucuronide (VIP score = 1.98). The Log_2_ fold‐change values for all VIP compounds compared with N are reported in Table 4, and those for the complete dataset in Table S1. To better visualize the metabolic impact of LAB fermentation, a heat map based on the 46 most discriminant VIP metabolites was generated (Figure 4). The heat map separated fermented samples from N, with most metabolites up‐accumulated in the inoculated treatments, particularly in BW and PBW. This pattern indicates that LAB fermentation modified the biochemical profile of banana pulp through both the release of endogenous phytochemicals and the formation of fermentation‐derived metabolites. Among the discriminant compounds, several phenolic and flavonoid‐related metabolites, including apigenin (highest Log_2_FC in W), esculetin (highest Log_2_FC in B), hydroxycaffeic acid (highest Log_2_FC in W), *p*‐coumaroyl malic acid (highest Log_2_FC in B and BW), sinensetin (highest Log_2_FC in B and P), gallagic acid (highest Log_2_FC in P), and ellagic acid derivatives (such as ellagic acid acetyl‐xyloside and ellagic acid glucoside), accumulated following fermentation. These changes likely derive from microbial esterase and glycosidase activities capable of hydrolyzing bound phenolics from the plant matrix and promoting their bioconversion into more soluble derivatives (Kumar et al. 2025). Similarly, the up accumulation of 5‐(3′,5′‐dihydroxyphenyl)‐γ‐valerolactone 3‐*O*‐glucuronide (particularly marked in BW and PBW) supports the occurrence of microbial catabolism of flavanol substrates during fermentation. Such modifications are relevant, as flavonoid derivatives have been reported to contribute to the antioxidant capacity of plant‐based matrices and may be involved in pathways linked to inflammatory modulation (Ferraz et al. 2020).

**TABLE 4:** Tabulated output of the supervised statistical analysis OPLS‐DA carried out on the most discriminant metabolites of the dataset. The calculations of the VIP score and the logarithmic fold change are also highlighted. The complete dataset is reported in Table S1.

Discriminating metabolites	VIP score	Log fold change						
		[P] vs [N]	[B] vs [N]	[W] vs [N]	[PB] vs [N]	[PW] vs [N]	[BW] vs [N]	[PBW] vs [N]
Spermine	1.111	−2.03	−1.65	0.47	0.62	−1.08	−2.48	−1.87
Thiamine (vitamin B1)	2.016	−1.02	−1.91	3.08	−2.57	2.68	2.71	2.74
Ascorbic acid	1.039	3.11	3.09	1.46	2.62	2.78	2.96	2.77
5‐(3′,5′‐dihydroxyphenyl)‐γ‐valerolactone 3‐*O*‐glucuronide	1.978	7.17	6.85	−0.28	5.82	5.41	7.68	7.73
3‐indole‐lactic acid	1.834	7.63	7.00	1.19	8.10	6.55	6.63	6.55
5‐methyltetrahydrofolic acid	1.461	−5.79	−6.66	−3.35	−5.87	−5.85	−6.80	−6.79

**FIGURE 4:**
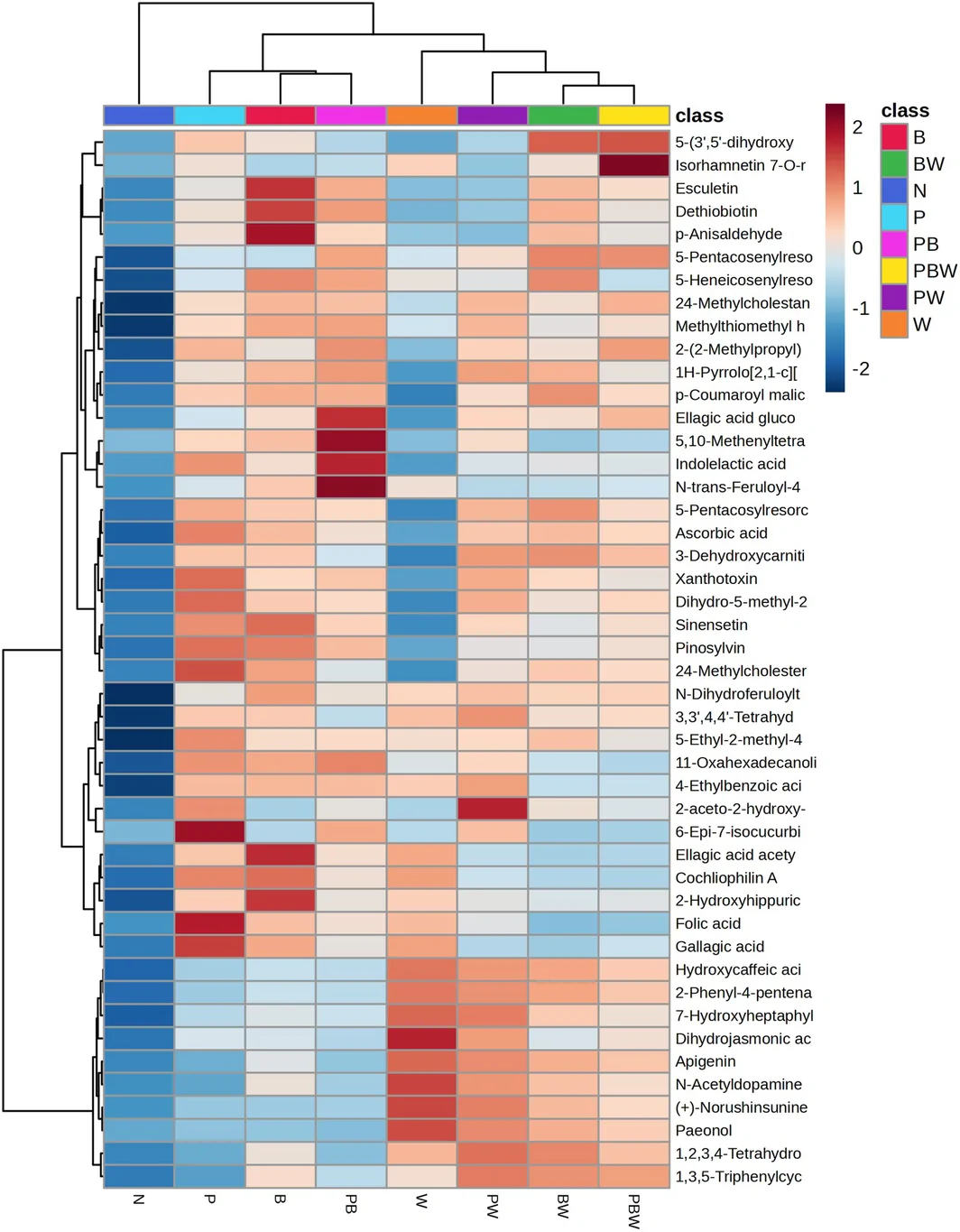
Heat map showing the only up‐accumulated metabolites (i.e., microbial fermentation products) when considering the different sample treatments.

Fermented samples (particularly PB) were also characterized by increased levels of 3‐indolelactic acid, a well‐known LAB‐derived metabolite originating from tryptophan metabolism (Roager and Licht 2018), together with folic acid, dethiobiotin, and 5,10‐methenyltetrahydrofolic acid, suggesting an active remodeling of vitamin‐related pathways during microbial growth. Fermentation was accompanied by a reduction in 5‐MTHF, one of the main biologically active folate forms, compared to the uninoculated control N. This decrease may be explained by bacterial uptake and intracellular folate cycling, together with the oxidative sensitivity of reduced folate vitamers during fermentation and sample handling (Homayouni Rad et al. 2016). In parallel, the higher relative abundance of ascorbic acid in the fermented samples is consistent with reduced oxidative degradation under the acidic conditions reached during fermentation (Capozzi et al. 2012; Hur et al. 2014). As no antioxidant assay was performed, this refers to metabolite abundance only. Ascorbic acid was not included among the classes subjected to semi‐quantification and was assessed only as relative abundance (VIP score = 1.04; Table S1). No absolute concentration is therefore available, and its contribution to reference intakes cannot be estimated from the present data. Moreover, banana pulp is not a relevant dietary source of vitamin C compared with a population reference intake of 95–110 mg/day for adults (EFSA 2013), and a relative increase in an already low baseline would not be expected to translate into a nutritionally meaningful amount. Several nitrogen‐ and sulfur‐containing metabolites, including N‐acetyldopamine, N‐dihydroferuloyltyramine, thiazolidine, and thiazole derivatives, were also enriched following fermentation. These compounds are likely associated with amino acid catabolism and secondary microbial transformations, potentially contributing to the development of the characteristic aroma complexity of fermented products. Additional discriminant metabolites showed strain‐dependent modulation patterns. For example, polyamine‐related compounds, such as spermine, decreased in most fermented samples while remaining closer to control levels in PB and W. Since polyamines are involved in cellular growth and intestinal homeostasis, these variations may reflect microbial utilization and metabolic turnover occurring during fermentation (Rao et al. 2020). Thiamine also displayed strain‐specific behavior, increasing in W while decreasing in the other treatments, suggesting differences in vitamin metabolism among microbial consortia.

Overall, LAB fermentation modified the metabolite profile of banana pulp relative to the spontaneously fermented control, with larger changes in the mixed‐culture treatments. The shifts observed in phenolic compounds, amino acid‐derived metabolites, ascorbic acid, and folate‐related metabolites were strain‐ and consortium‐dependent.

## 4 Conclusions

This study demonstrated that controlled lactic fermentation can modify the composition of banana pulp and convert it into a freeze‐dried ingredient with a reduced load of *Enterobacteriaceae*. The three tested strains showed distinct technological and metabolic behaviors, each contributing differently to the final product. *L. plantarum* UC9052 fermented the broadest range of carbohydrates and produced the fastest acidification, reaching the lowest pH within 24 h and the highest cell densities, with an earlier reduction of *Enterobacteriaceae*. *W. cibaria* UC9053 was the only strain producing exopolysaccharides, a trait relevant to the texture and water‐holding properties of fermented matrices, but its slower acidification resulted in a later reduction of *Enterobacteriaceae*, which were still detectable at 24 h, and in a metabolomic profile closer to that of the spontaneously fermented control. *L. brevis* UC9051, although less versatile in sugar utilization, contributed to the modulation of phenolic‐related metabolites, particularly when combined with *L. plantarum* . The three strains showed comparable antifungal activity (*p* = 0.257, one‐way ANOVA). Among the tested formulations, the mixed cultures combined the technological and metabolic contributions of the individual strains. The binary consortium PB ( *L. plantarum* + *L. brevis* ) combined the rapid acidification driven by *L. plantarum* with the highest cell density at 24 h. Although fermentation generally reduced folate and flavonol levels, PB showed the smallest change with respect to the spontaneously fermented control (N) (folates 106.6 vs. 106.3 μg Eq./100 g; flavonols 85.0 vs. 92.9 μg Eq./100 g), together with the highest accumulation of 3‐indolelactic acid, a tryptophan‐derived metabolite of microbial origin. The ternary consortium PBW reached the highest flavanol content among all samples, including the spontaneously fermented control (N) (105.2 vs. 65.3 μg Eq./100 g). All the above values are semi‐quantitative, expressed as equivalents of the corresponding reference standard. On this basis, two complementary applications can be proposed: PB as a balanced starter formulation when the priority is rapid acidification combined with retention of bioactive compounds, and PBW when the goal is to obtain higher levels of specific phenolic classes such as flavanols. Freeze‐drying of the fermented purée preserved viable LAB counts between 6.93 and 7.91 log_10_ CFU/mL across all formulations, with comparable survival rates between single and mixed cultures. Further work is needed to evaluate the technological behavior of the freeze‐dried ingredient in real food applications (e.g., bakery and fruit‐based products), to assess sensory acceptability, and to perform a more comprehensive nutritional and shelf‐life characterization. The optimization of fermentation parameters at pilot scale and the use of cryoprotectants could further improve the survival rates and metabolic stability of the selected starter consortium.

## Author Contributions

**Sara Morandi:** conceptualization, methodology, investigation, data curation, formal analysis, writing – original draft, software. **Gabriele Rocchetti:** methodology, investigation, formal analysis, visualization, writing – review and editing, software. **Claudia Cortimiglia:** methodology, writing – review and editing, validation. **Daniela Bassi:** conceptualization, supervision, funding acquisition, resources, writing – review and editing, project administration.

## Funding

This work was supported by the project funded under National Recovery and Resilience Plan (NRRP), Mission 4 Component 2 Investment 1.3‐Call for proposals No. 341 of 15 March 2022 of the Italian Ministry of University and Research funded by the European Union‐NextGenerationEU (Award Number: Project code PE00000003, Concession Decree No. 1550 of 11 October 2022 adopted by the Italian Ministry of University and Research, CUP D93C22000890001, Project title “ON Foods‐Research and innovation network on food and nutrition Sustainability, Safety and Security‐Working ON Foods”). Additional support was provided by the PhD programme in Agro‐Food Systems (AgriSystem) and by the Portus project, funded by the Romeo and Enrica Invernizzi Foundation.

## Ethics Statement

The authors have nothing to report.

## Consent

The authors have nothing to report.

## Conflicts of Interest

The authors declare no conflicts of interest.

## Supporting information


**Table S1:** Untargeted metabolomic characterization of fermented banana pulp samples. Sheet 1: complete UHPLC‐HRMS dataset with metabolite annotation and raw abundance values. Sheet 2: semi‐quantitative analysis of the main bioactive classes, expressed as μg equivalents/100 g, with post hoc groupings. Sheet 3: OPLS‐DA model parameters and Log_2_ fold‐change values of the VIP markers.

## Data Availability

The data that support the findings of this study are available from the corresponding author upon reasonable request.

## References

[fsn372577-bib-0001] Aboul‐Enein, A. , Z. Salama , A. A. Gaafar , and H. H. Aly . 2016. “Identification of Phenolic Compounds From Banana Peel (*Musa paradaisica* L.) as Antioxidant and Antimicrobial Agents Egyptian Patients With Autism Spectrum Disorder View Project MSc in Biochemistry View Project.” https://www.researchgate.net/publication/302118414.

[fsn372577-bib-0002] Adesulu‐Dahunsi, A. T. , A. I. Sanni , and K. Jeyaram . 2018. “Production, Characterization and In Vitro Antioxidant Activities of Exopolysaccharide From *Weissella cibaria* GA44.” LWT 87: 432–442. 10.1016/j.lwt.2017.09.013.

[fsn372577-bib-0003] Arena, M. P. , A. Silvain , G. Normanno , et al. 2016. “Use of *Lactobacillus plantarum* Strains as a Bio‐Control Strategy Against Food‐Borne Pathogenic Microorganisms.” Frontiers in Microbiology 7: 1–10. 10.3389/fmicb.2016.00464. PMC4829616 27148172

[fsn372577-bib-0004] Aronson, J. K. 2017. “Defining ‘Nutraceuticals’: Neither Nutritious nor Pharmaceutical.” British Journal of Clinical Pharmacology 83, no. 1: 8–19. 10.1111/bcp.12935. PMC5338166 26991455

[fsn372577-bib-0005] Besrour‐Aouam, N. , I. Fhoula , A. M. Hernández‐Alcántara , et al. 2021. “The Role of Dextran Production in the Metabolic Context of Leuconostoc and Weissella Tunisian Strains.” Carbohydrate Polymers 253: 117254. 10.1016/j.carbpol.2020.117254. 33279004

[fsn372577-bib-0006] Bhola, J. , and R. Bhadekar . 2024. “Prebiotic Effect of Daily Dietary Polyphenols and Oligosaccharides on Lactobacillus Species.” Bioactive Carbohydrates and Dietary Fibre 31: 100407. 10.1016/j.bcdf.2024.100407.

[fsn372577-bib-0007] Bozzetti, M. , C. Cerri , S. Morandi , et al. 2025. “Untargeted Screening Based on UHPLC‐HRMS of Total Folates Produced by Lactic Acid Bacteria in Fermented Milk and During Yogurt Shelf Life.” Food 14, no. 7: 1112. 10.3390/foods14071112. PMC11989110 40238192

[fsn372577-bib-0062] Capozzi, V. , P. Russo , M. T. Dueñas , P. López , and G. Spano . 2012. “Lactic Acid Bacteria Producing B‐Group Vitamins: A Great Potential for Functional Cereals Products.” Applied Microbiology and Biotechnology 96, no. 6: 1383–1394. 10.1007/s00253-012-4440-2. 23093174

[fsn372577-bib-0008] Chiarini, E. , V. Alessandria , D. Buzzanca , et al. 2024. “Valorization of Fruit by‐Products Through Lactic Acid Fermentation for Innovative Beverage Formulation: Microbiological and Physiochemical Effects.” Food 13, no. 23: 3715. 10.3390/foods13233715. PMC11640541 39682787

[fsn372577-bib-0009] Cui, S. , M. Hu , Y. Sun , et al. 2022. “Effect of Trehalose and Lactose Treatments on the Freeze‐Drying Resistance of Lactic Acid Bacteria in High‐Density Culture.” Microorganisms 11, no. 1: 48. 10.3390/microorganisms11010048. PMC9866448 36677339

[fsn372577-bib-0010] Cui, S. , Z. Pan , S. Wu , et al. 2022. “Improvement of the Lyophilization Survival Rate of *Lactobacillus casei* via Regulation of Its Surface Substances.” Food 11, no. 21: 3468. 10.3390/foods11213468. PMC9656813 36360081

[fsn372577-bib-0060] Czarnowska‐Kujawska, M. , and B. Paszczyk . 2021. “Changes in the Folate Content and Fatty Acid Profile in Fermented Milk Produced With Different Starter Cultures During Storage.” Molecules 26, no. 19: 6063. 10.3390/molecules26196063. PMC8512886 34641607

[fsn372577-bib-0011] Dal Bello, F. , C. I. Clarke , L. A. M. Ryan , et al. 2007. “Improvement of the Quality and Shelf Life of Wheat Bread by Fermentation With the Antifungal Strain *Lactobacillus plantarum* FST 1.7.” Journal of Cereal Science 45, no. 3: 309–318. 10.1016/j.jcs.2006.09.004.

[fsn372577-bib-0012] Dalié, D. K. D. , A. M. Deschamps , and F. Richard‐Forget . 2010. “Lactic Acid Bacteria‐Potential for Control of Mould Growth and Mycotoxins: A Review.” Food Control 21, no. 4: 370–380. 10.1016/j.foodcont.2009.07.011.

[fsn372577-bib-0013] Di Cagno, R. , M. De Angelis , A. Limitone , et al. 2006. “Glucan and Fructan Production by Sourdough Weissella Cibaria and *Lactobacillus plantarum* .” Journal of Agricultural and Food Chemistry 54, no. 26: 9873–9881. 10.1021/jf061393+. 17177514

[fsn372577-bib-0014] Du, R. , L. Yu , N. Yu , W. Ping , G. Song , and J. Ge . 2022. “Characterization of Exopolysaccharide Produced by Levilactobacillus Brevis HDE‐9 and Evaluation of Its Potential Use in Dairy Products.” International Journal of Biological Macromolecules 217: 303–311. 10.1016/j.ijbiomac.2022.07.057. 35839950

[fsn372577-bib-0015] EFSA . 2013. “Scientific Opinion on Dietary Reference Values for Vitamin C.” EFSA Journal 11, no. 11: 3418. 10.2903/j.efsa.2013.3418.

[fsn372577-bib-0016] EFSA . 2014. “Scientific Opinion on Dietary Reference Values for Folate.” EFSA Journal 12, no. 11: 3893. 10.2903/j.efsa.2014.3893.

[fsn372577-bib-0017] Fekete, M. , A. Lehoczki , A. Kryczyk‐Poprawa , et al. 2025. “Functional Foods in Modern Nutrition Science: Mechanisms, Evidence, and Public Health Implications.” In Nutrients, vol. 17, 13. Multidisciplinary Digital Publishing Institute (MDPI). 10.3390/nu17132153. PMC12251440 40647258

[fsn372577-bib-0018] Ferraz, C. R. , T. T. Carvalho , M. F. Manchope , et al. 2020. “Therapeutic Potential of Flavonoids in Pain and Inflammation: Mechanisms of Action, Pre‐Clinical and Clinical Data, and Pharmaceutical Development.” Molecules 25, no. 3: 762. 10.3390/molecules25030762. PMC7037709 32050623

[fsn372577-bib-0019] Fusco, V. , G. M. Quero , G.‐S. Cho , et al. 2015. “The Genus Weissella: Taxonomy, Ecology and Biotechnological Potential.” Frontiers in Microbiology 6: 155. 10.3389/fmicb.2015.00155. PMC4362408 25852652

[fsn372577-bib-0020] Gallo, M. , F. Passannanti , P. Schiattarella , et al. 2021. “Banana Puree Lactic Fermentation: The Role of Ripeness, Heat Treatment, and Ascorbic Acid.” Applied Sciences 11, no. 11: 5153. 10.3390/app11115153.

[fsn372577-bib-0021] Garcia, C. , M. Guerin , K. Souidi , and F. Remize . 2020. “Lactic Fermented Fruit or Vegetable Juices: Past, Present and Future.” Beverages 6, no. 1: 8. 10.3390/beverages6010008.

[fsn372577-bib-0022] Gomes, J. F. S. , R. R. Vieira , and F. R. Leta . 2013. “Colorimetric Indicator for Classification of Bananas During Ripening.” Scientia Horticulturae 150: 201–205. 10.1016/j.scienta.2012.11.014.

[fsn372577-bib-0023] Guérin, M. , C. R.‐D. Silva , C. Garcia , and F. Remize . 2020. “Lactic Acid Bacterial Production of Exopolysaccharides From Fruit and Vegetables and Associated Benefits.” Fermentation 6, no. 4: 115. 10.3390/fermentation6040115.

[fsn372577-bib-0024] Hacıoglu, S. , and B. Kunduhoglu . 2021. “Probiotic Characteristics of *Lactobacillus brevis* KT38‐3 Isolated From an Artisanal Tulum Cheese.” Food Science of Animal Resources 41, no. 6: 967–982. 10.5851/kosfa.2021.e49. PMC8564325 34796324

[fsn372577-bib-0025] Hashim, M. 2023. “Functional, Nutritional and Medicinal Potential of Banana Peel.” Pure and Applied Biology 12, no. 1: 120049. 10.19045/bspab.2023.120049.

[fsn372577-bib-0061] Homayouni Rad, A. , A. Yari Khosroushahi , M. Khalili , and S. Jafarzadeh . 2016. “Folate Bio‐Fortification of Yoghurt and Fermented Milk: A Review.” Dairy Science & Technology 96, no. 4: 427–441. 10.1007/s13594-016-0286-1.

[fsn372577-bib-0026] Huang, P.‐H. , Y.‐T. Cheng , W.‐C. Lu , et al. 2024. “Changes in Nutrient Content and Physicochemical Properties of Cavendish Bananas Var. Pei Chiao During Ripening.” Horticulturae 10, no. 4: 384. 10.3390/horticulturae10040384.

[fsn372577-bib-0027] Hui, Y. H. , and E. Ö. Evranuz . 2012. “Fermentation and Biopreservation of Plant‐ Based Foods With Lactic Acid Bacteria.” In Handbook of Plant‐Based Fermented Food and Beverage Technology. CRC Press. 10.1201/b12055.

[fsn372577-bib-0064] Hur, S. J. , S. Y. Lee , Y.‐C. Kim , I. Choi , and G.‐B. Kim . 2014. “Effect of Fermentation on the Antioxidant Activity in Plant‐Based Foods.” Food Chemistry 160: 346–356. 10.1016/j.foodchem.2014.03.112. 24799248

[fsn372577-bib-0028] Kumar, A. , S. Saranyadevi , S. K. Thirumalaisamy , et al. 2025. “Phenolic Acids in Fermented Foods: Microbial Biotransformation, Antioxidant Mechanisms, and Functional Health Implications.” Frontiers in Molecular Biosciences 12: 1678673. 10.3389/fmolb.2025.1678673. PMC12583000 41195418

[fsn372577-bib-0029] Kumari, A. , A. Agarwal , A. Aggarwal , et al. 2022. “Scope of Banana by‐Products: A Potent Human Resource.” International Journal of Current Microbiology and Applied Sciences 11, no. 9: 104–112. 10.20546/ijcmas.2022.1109.012.

[fsn372577-bib-0030] Lahiri, D. , M. Nag , T. Sarkar , et al. 2022. “Lactic Acid Bacteria (LAB): Autochthonous and Probiotic Microbes for Meat Preservation and Fortification.” Food 11, no. 18: 2792. 10.3390/foods11182792. PMC9498044 36140920

[fsn372577-bib-0031] Leroy, F. , and L. De Vuyst . 2004. “Lactic Acid Bacteria as Functional Starter Cultures for the Food Fermentation Industry.” Trends in Food Science & Technology 15, no. 2: 67–78. 10.1016/j.tifs.2003.09.004.

[fsn372577-bib-0032] Lizana, L. A. 1976. “Quantitative Evolution of Sugars in Banana Fruit Ripening at Normal to Elevated Temperatures.” Acta Horticulturae 57: 163–178. 10.17660/ActaHortic.1976.57.20.

[fsn372577-bib-0033] Lu, Y. , S. Song , H. Tian , H. Yu , J. Zhao , and C. Chen . 2018. “Functional Analysis of the Role of CcpA in *Lactobacillus plantarum* Grown on Fructooligosaccharides or Glucose: A Transcriptomic Perspective.” Microbial Cell Factories 17, no. 1: 201. 10.1186/s12934-018-1050-4. PMC6309078 30593274

[fsn372577-bib-0034] Mia, M. S. , M. M. Ahmed , and W. Zzaman . 2025. “Valorization of Food Waste Into Functional Ingredients Supports a Sustainable Strategy for the Food Industry.” Discover Food 5, no. 1: 275. 10.1007/s44187-025-00584-3.

[fsn372577-bib-0035] Mohd Zaini, H. , J. Roslan , S. Saallah , E. Munsu , N. S. Sulaiman , and W. Pindi . 2022. “Banana Peels as a Bioactive Ingredient and Its Potential Application in the Food Industry.” Journal of Functional Foods 92: 105054. 10.1016/j.jff.2022.105054.

[fsn372577-bib-0036] Muhammad Ansari, N. A. I. , N. Ramly , N. H. Faujan , and N. Arifin . 2023. “Nutritional Content and Bioactive Compounds of Banana Peel and Its Potential Utilization: A Review.” Malaysian Journal of Science Health & Technology 9, no. 1: 74–86. 10.33102/mjosht.v9i1.313.

[fsn372577-bib-0037] Muhialdin, B. J. , Z. Hassan , and N. Saari . 2018. “In Vitro Antifungal Activity of Lactic Acid Bacteria Low Molecular Peptides Against Spoilage Fungi of Bakery Products.” Annals of Microbiology 68, no. 9: 557–567. 10.1007/s13213-018-1363-x.

[fsn372577-bib-0038] Nguyen, V.‐L. , N.‐N. Nguyen , A. D. Do , T.‐S. Nguyen , and Q.‐D. Nguyen . 2024. “Exopolysaccharides From *Weissella cibaria* Isolated From Oats: Isolation, Characterisation and Its Application in Improving the Texture of Set‐Type Yogurt.” International Journal of Food Science and Technology 59, no. 12: 9532–9546. 10.1111/ijfs.17603.

[fsn372577-bib-0039] Ramos, C. L. , L. Thorsen , R. F. Schwan , and L. Jespersen . 2013. “Strain‐Specific Probiotics Properties of *Lactobacillus fermentum* , Lactobacillus Plantarum and *Lactobacillus brevis* Isolates From Brazilian Food Products.” Food Microbiology 36, no. 1: 22–29. 10.1016/j.fm.2013.03.010. 23764216

[fsn372577-bib-0063] Rao, J. N. , L. Xiao , and J.‐Y. Wang . 2020. “Polyamines in Gut Epithelial Renewal and Barrier Function.” Physiology 35, no. 5: 328–337. 10.1152/physiol.00011.2020. PMC7642847 32783609

[fsn372577-bib-0040] Râpă, M. , R. N. Darie‐Niță , and G. Coman . 2024. “Valorization of Fruit and Vegetable Waste Into Sustainable and Value‐Added Materials.” Waste 2, no. 3: 258–278. 10.3390/waste2030015.

[fsn372577-bib-0041] Roager, H. M. , and T. R. Licht . 2018. “Microbial Tryptophan Catabolites in Health and Disease.” Nature Communications 9, no. 1: 3294. 10.1038/s41467-018-05470-4. PMC6098093 30120222

[fsn372577-bib-0042] Rocchetti, G. , M. Lapris , G. Zengin , F. Masoero , and A. Gallo . 2024. “Discrimination of Different Feed Additives and Poly‐Herbal Formulations Based on Their Untargeted Phytochemical Profiles.” Metabolomics 20, no. 1: 5. 10.1007/s11306-023-02068-w.

[fsn372577-bib-0043] Rocchetti, G. , and T. F. O'Callaghan . 2021. “Application of Metabolomics to Assess Milk Quality and Traceability.” Current Opinion in Food Science 40: 168–178. 10.1016/j.cofs.2021.04.005.

[fsn372577-bib-0044] Rönkä, E. , E. Malinen , M. Saarela , M. Rinta‐Koski , J. Aarnikunnas , and A. Palva . 2003. “Probiotic and Milk Technological Properties of *Lactobacillus brevis* .” International Journal of Food Microbiology 83, no. 1: 63–74. 10.1016/S0168-1605(02)00315-X. 12672593

[fsn372577-bib-0045] Sabater, C. , L. Ruiz , S. Delgado , P. Ruas‐Madiedo , and A. Margolles . 2020. “Valorization of Vegetable Food Waste and by‐Products Through Fermentation Processes.” Frontiers in Microbiology 11: 1–11. 10.3389/fmicb.2020.581997. PMC7606337 33193217

[fsn372577-bib-0046] Sam, F. E. , and A. N. A. Aryee . 2026. “Fermented Plant‐Based Foods: Insights and Emerging Trends.” Food Research International 245: 120536. 10.1016/j.foodres.2026.120536. 42914298

[fsn372577-bib-0047] Siezen, R. J. , and J. E. van Hylckama Vlieg . 2011. “Genomic Diversity and Versatility of *Lactobacillus plantarum* , a Natural Metabolic Engineer.” Microbial Cell Factories 10, no. Suppl 1: S3. 10.1186/1475-2859-10-S1-S3. PMC3271238 21995294

[fsn372577-bib-0048] Souza, L. V. , R. Rodrigues da Silva , A. Falqueto , et al. 2023. “Evaluation of Antifungal Activity of Lactic Acid Bacteria Against Fungi in Simulated Cheese Matrix.” LWT 182: 114773. 10.1016/j.lwt.2023.114773.

[fsn372577-bib-0049] Tan, M. , X. Yan , H. Ramaswamy , and C. Wang . 2025. “Mapping Biosynthesis Pathway of Natural Folate During Lactiplantibacillus Plantarum Subsp. Plantarum Fermentation in Purple Cabbage by Transcriptomic Analysis.” Journal of the Science of Food and Agriculture 105, no. 5: 2854–2866. 10.1002/jsfa.14053. 39614591

[fsn372577-bib-0050] Tang, H.‐J. , C.‐C. Chen , Y.‐C. Lu , et al. 2022. “The Effect of Lactobacillus With Prebiotics on KPC‐2‐Producing *Klebsiella pneumoniae* .” Frontiers in Microbiology 13: 1050247. 10.3389/fmicb.2022.1050247. PMC9767986 36569071

[fsn372577-bib-0051] Tebyanian, H. , A. Bakhtiari , A. Karami , and A. Kariminik . 2017. “Antimicrobial Activity of Some Lactobacillus Species Against Intestinal Pathogenic Bacteria.” International Letters of Natural Sciences 65: 10–15. 10.18052/www.scipress.com/ILNS.65.10.

[fsn372577-bib-0052] Thompson, H. O. , G. Önning , K. Holmgren , H. S. Strandler , and M. Hultberg . 2020. “Fermentation of Cauliflower and White Beans With *Lactobacillus plantarum* – Impact on Levels of Riboflavin, Folate, Vitamin B12, and Amino Acid Composition.” Plant Foods for Human Nutrition 75, no. 2: 236–242. 10.1007/s11130-020-00806-2. PMC7266841 32144644

[fsn372577-bib-0053] Tsugawa, H. , T. Cajka , T. Kind , et al. 2015. “MS‐DIAL: Data‐Independent MS/MS Deconvolution for Comprehensive Metabolome Analysis.” Nature Methods 12, no. 6: 523–526. 10.1038/nmeth.3393. PMC4449330 25938372

[fsn372577-bib-0054] Yang, X. , J. Hong , L. Wang , et al. 2024. “Effect of Lactic Acid Bacteria Fermentation on Plant‐Based Products.” Fermentation 10, no. 1: 48. 10.3390/fermentation10010048.

[fsn372577-bib-0055] Yap, M. , W. M. A. D. B. Fernando , C. S. Brennan , V. Jayasena , and R. Coorey . 2017. “The Effects of Banana Ripeness on Quality Indices for Puree Production.” LWT 80: 10–18. 10.1016/j.lwt.2017.01.073.

[fsn372577-bib-0056] Yu, A. O. , J. H. J. Leveau , and M. L. Marco . 2020. “Abundance, Diversity and Plant‐Specific Adaptations of Plant‐Associated Lactic Acid Bacteria.” Environmental Microbiology Reports 12, no. 1: 16–29. 10.1111/1758-2229.12794. 31573142

[fsn372577-bib-0057] Zapaśnik, A. , B. Sokołowska , and M. Bryła . 2022. “Role of Lactic Acid Bacteria in Food Preservation and Safety.” Food 11, no. 9: 1–17. 10.3390/foods11091283. PMC9099756 35564005

[fsn372577-bib-0058] Zhou, K. , Y. Zeng , M. Yang , et al. 2016. “Production, Purification and Structural Study of an Exopolysaccharide From *Lactobacillus plantarum* BC‐25.” Carbohydrate Polymers 144: 205–214. 10.1016/j.carbpol.2016.02.067. 27083810

[fsn372577-bib-0059] Zhu, Y. , C. Wang , S. Jia , et al. 2018. “Purification, Characterization and Antioxidant Activity of the Exopolysaccharide From *Weissella cibaria* SJ14 Isolated From Sichuan Paocai.” International Journal of Biological Macromolecules 115: 820–828. 10.1016/j.ijbiomac.2018.04.067. 29660455

